# Aging, Social Distancing, and COVID-19 Risk: Who is more Vulnerable and Why?

**DOI:** 10.14336/AD.2021.0319

**Published:** 2021-10-01

**Authors:** Jamshid Faraji, Gerlinde A.S Metz

**Affiliations:** ^1^Canadian Centre for Behavioural Neuroscience, University of Lethbridge, Lethbridge, AB, Canada; ^2^Faculty of Nursing & Midwifery, Golestan University of Medical Sciences, Gorgan, Iran

**Keywords:** COVID-19, novel coronavirus, social distancing, physical distancing, lockdown, confinement, social isolation, senior care, nursing homes, aging, two-hit model, allostasis, allostatic load

## Abstract

Perceived social support represents an important predictor of healthy aging. The global COVID-19 pandemic has dramatically changed the face of social relationships and revealed elderly to be particularly vulnerable to the effects of social isolation. Social distancing may represent a double-edged sword for older adults, protecting them against COVID-19 infection while also sacrificing personal interaction and attention at a critical time. Here, we consider the moderating role of social relationships as a potential influence on stress resilience, allostatic load, and vulnerability to infection and adverse health outcomes in the elderly population. Understanding the mechanisms how social support enhances resilience to stress and promotes mental and physical health into old age will enable new preventive strategies. Targeted social interventions may provide effective relief from the impact of COVID-19-related isolation and loneliness. In this regard, a pandemic may also offer a window of opportunity for raising awareness and mobilizing resources for new strategies that help build resilience in our aging population and future generations.

The novel coronavirus disease 2019, SARS-CoV-2 or COVID-19, is now recognized as one of the most contagious human viral infections. It gave rise to a global pandemic and a global health emergency with dramatic, still unpredictable social and economic long-term consequences for our society. Elderly individuals seem to be particularly vulnerable to developing severe COVID-19 infection, with higher risk of morbidity and mortality than any other age group [1, 2]. Fatalities in the general population were significantly reduced by rapid social engineering and early preventative policies imposed by governments and health officials around the world, including travel restrictions, curfews and social-physical distancing [3, 4]. Nevertheless, population-based patterns of adverse health outcomes from COVID-19 still reflect greater vulnerability in the elderly, and in long-term senior care facilities and nursing homes [5, 6].

The past century has seen an extension in life expectancy globally, almost doubling the average lifespan in Western countries [7]. The global aging population is rapidly growing and in 2020 the number of individuals 60 years and older for the first time is expected to exceed the number of children and youth [8]. Increased longevity is not necessarily associated with an extended healthspan, however, due to adverse cultural and lifestyle determinants and poor environmental conditions [9].The definition of aging refers to a time-dependent functional decline that is typically accompanied by progressive deterioration of physiological and cellular integrity. For example, about 88% of North Americans aged 65 years and older experience significant physical and mental health decline [10] which results from accumulated cell and DNA damage acquired across the lifespan [10, 11], raising the risk of immune deficiencies, poor cardiovascular outcomes and non-communicable disease risk, cognitive and neurological impairments, and mobility problems [11]. It is therefore not unexpected that older adults are generally at greater risk of COVID-19 infection than other age groups (https://www.who.int).

Acceleration in the biological processes of cellular aging, experiential, environmental and pathological factors may raise individual vulnerability to COVID-19 infection and severe symptoms. This is especially true for elderly individuals with chronic medical conditions like diabetes as they are reportedly at higher risk of developing severe COVID-19 complications [5, 12]. Based on reports of non-communicable and communicable diseases, it is possible that COVID-19 vulnerability includes adverse childhood and lifetime experiences [13], heritable epigenetic markers linked to ancestral stress [14-16], environmental pollution [17, 18], poor diet [19], and poor social support [20-22]. Each of these factors may be considered a stressor, or a “hit”, with potentially cumulative impacts [23, 24] on COVID-19 vulnerability and complication. Across a single lifespan, the effects of recurrent stress may accumulate [25], thus increasing the body’s “wear and tear” and allostatic load (AL), ultimately heightening the vulnerability to disease in an older individual [26, 27]. This framework has been well operationalized to explain the origins of complex diseases, including cardiovascular [28], kidney [29], lung [30], psychiatric and neurological [31-35] diseases. Experiences in older age, particularly by social experiences, add another important layer to these allostatic load considerations.

The intensity of social relationships in Western societies is generally diminishing, and elderly are particularly concerned by social isolation and feelings of loneliness [36, 37]. Although the definition of social isolation and loneliness varies, biological sex and gender are consistent key determinants of their prevalence in population-based surveys. If isolation is defined as living alone, approximately 20% of elderly men and nearly 40% of elderly women in Western societies report that they feel isolated and lack social contacts [38]. Lack of social relationships can instigate or exacerbate stress responses [39], depression and anxiety [20] and reduce the lifespan [40-42]. Individuals with weaker social relationships are 50% less likely to survive a health issue, regardless of age, initial health status or cause of death [43].

The nature of social relationships dramatically changes as individuals age, however. Social networks tend to narrow with increasing age, and novelty seeking, which facilitates building new social relationships, becomes reduced in old age [43]. Hence, the loss of peripheral members of social networks is larger than the loss of intimate members, although both contribute to the well-being of older adults. Despite a shrinking social network during aging, the need for proximity, meaningful relationships and reciprocity [44] are characteristic features of social needs in late life.

The role of social experiences in health outcomes demands particular consideration with respect to the COVID-19 pandemic. During the COVID-19 pandemic, physical distancing was introduced as an effective prevetive measure to reduce the risk of infection [45]. While this has significantly helped to contain the global spread of infection, it has also exacerbated the social isolation of elderly as visits of family and friends to nursing homes and long-term care facilities were prohibited to protect their residents. At all ages, the COVID-19 pandemic has increased the prevalence of negative psychological effects such as post-traumatic stress disorders (PTSD), confusion, and anger due to quarantine, fear, frustration, boredom, inadequate financial resources and supplies, and stigma [46]. PTSD, which is mainly caused by severe and traumatic stress, is associated with accelerated aging and telomere length (TL) erosion [47], a cellular index of premature senescence. In mammals, telomeres protect genetic material from degradation during somatic cell division. Because the TL decreases along with proliferation in each normal cell division, telomere shortening can be considered a measure of biological aging [48]. More importantly, PTSD in the elderly and its extensive impact on aging process follow a complex clinical trajectory that present unique aspects not seen in young populations [49]. Therefore, elderly individuals are particularly vulnerable to the adverse impact of stressors and traumatic experiences such as social isolation that could be exacerbated by the pandemic. Furthermore, social frailty as a non-specific state of vulnerability in older people has recently received attention in the context of psychological disturbances induced by the COVID-19-related isolation measures [50], because there is evidence that social engagement is an indicator of successful aging.

Here, we will consider the moderating role of social relationships as a potential influence on COVID-19 vulnerability and adverse outcomes in the elderly population. The presented literature will elucidate the hypothesis that resilience of the host to SARS-CoV-2 infection and associated complications will be reduced by cumulative lifetime stress exposure. Considering that lifetime exposure to various stresses may represent the first “hit”, social isolation in older age may exacerbate these effects as a second “hit” and generate a potentially harmful stress burden. We will discuss the underlying physiological mechanisms of psychosocial stress and integrate the existing literature to propose a unifying concept of the social determinants of healthy aging and potential mitigation strategies.

## Social interaction: the precursor of psychobiological integrity

Humans did not evolve to be alone [51]. Influenced by Aristotle’s famous aphorism, it is frequently being posited that, by nature, we are social animals, tautologically meaning that we tend to live with others and enjoy social interactions because we are inherently social beings. Reflections of social life in human biology and brain development [52-54], however, go beyond the limits of such equivocal notions. In fact, humans *interact* with others across the lifespan, and at the same time also *influence* others while accepting influences from them. This reciprocal influence of social life brings about changes at different levels (i.e., attitudes, motivations, beliefs and behaviours) [55]. The human brain is highly attuned to social interactions between multiple elements [56], and evolved to perceive and appraise social interactions already starting during prenatal development [57], reflecting biological adaptation [58]. Social interactions occur along a continuum ranging from full engagement in social communication to a complete disengagement from social ties (social isolation) and supports. Social supports, in theoretical models, encompass two important quantitative and qualitative dimensions. Quantitative (structural) aspects of social support include social interaction, which is measured in terms of the quantity of friends and relatives as well as frequency of social interactions an individual report. Social support also can be measured qualitatively or functionally through evaluating the quality of emotional (receiving love, acceptance and empathy) or instrumental (practical help) supports [59]. Both components of social interaction are reflected by objective biological parameters of health and longevity [60].

### Quality and Quantity of Social Interactions in Aging

Psychological processes that contribute to healthy aging via social interactions appear to exert protective functions against maladaptive physical disease states [40, 41]. Although a detailed review of research over the impact of the quality and quantity of social life in the elderly is outside the scope of this perspective, two important points need to be addressed. First, social relationships, depending upon their cultural determinants and conceptualization, are multidimensional, and thus their links to health (particularly in senescence) are multifaceted. Since the impact of social interaction on health outcomes is generally dependent upon multiple layers of society (e.g. family, friends, colleagues, organizations, and community) [61], there is continuous need for extensive policy reform or reconceptualization aimed at improving social atmosphere and systems for aging populations. Second, social interaction *per se* does not necessarily guarantee health promotion. Investigations are mainly focusing on understanding and characterizing the dynamics underlying *positive* social interaction whereas its *negative* face is largely ignored, particularly by mainstream gerontological research. Negative interactions may entail unpleasant social encounters that are characterized by the lack of reciprocity, criticism and rejection [62], or discouraging the expression of feeling and failing to provide promised help [63]. Although both aversive and positive (facilitating) social interactions are fundamental influences on brain and behaviour [64], arguably only meaningful positive social interactions impart benefits for health.

Positive interactions between individuals in a social network [65] may improve the quality of life and alleviate existing health complications. Potential mitigating mechanisms include reduced pro-inflammatory processes [66] and dampened hormonal stress responses [67] along with other changes in biophysiological mechanisms [68]. Psychologically, social integration provides informational and emotional resources that promote adaptive behavioural responses (e.g. self-disclosure) to stressful experiences such as diseases and trauma [43], thus building resilience and buffering their deleterious impact on health, or shaping health behaviours [69]. It seems that when these psychosocial resources are available, individuals experience an enriched opportunity to reciprocally share emotions and optimize their coping skills. Sharing the emotional burden of stress may provide relief for the afflicted, but it may also generate bystander stress for their receiving social contacts [70].

### Social Needs and Sex Differences in Aging

**Figure 1.**
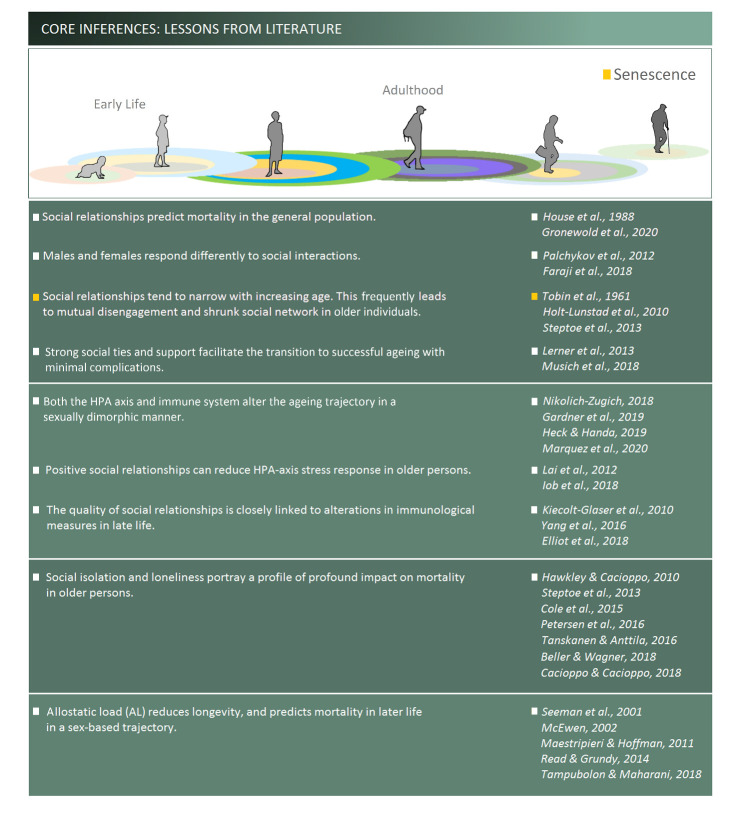
Overview of relevant literature that discussed link social support, stress response and/or immune status to the chances of healthy aging.

The nature of social relationships changes during the lifespan. Aging in particular may be accompanied by social disengagement or withdrawal [71] driven by the hegemony of emerging new needs and priorities. The need for a new equilibrium between the social needs of an aging individual and their environment may gradually alter the existing social relationship [72] and may result in greater physical distance than what existed in the middle age [73]. Accordingly, social ties in aging adults are closely linked to a variety of challenges such as physical disabilities [74-76], chronic neurodegenerative diseases [77, 78], health risks [79-81], negative psychological states [79, 82, 83], as well as morbidity and mortality [84-86]. Consequently, the nature of social relationships in aged individuals may predict mortality [43, 87, 88]. As summarized in Figure 1, a strong social network and available support, alternatively, provides adults with the prospect of successful aging [89, 90].

**Figure 2.**
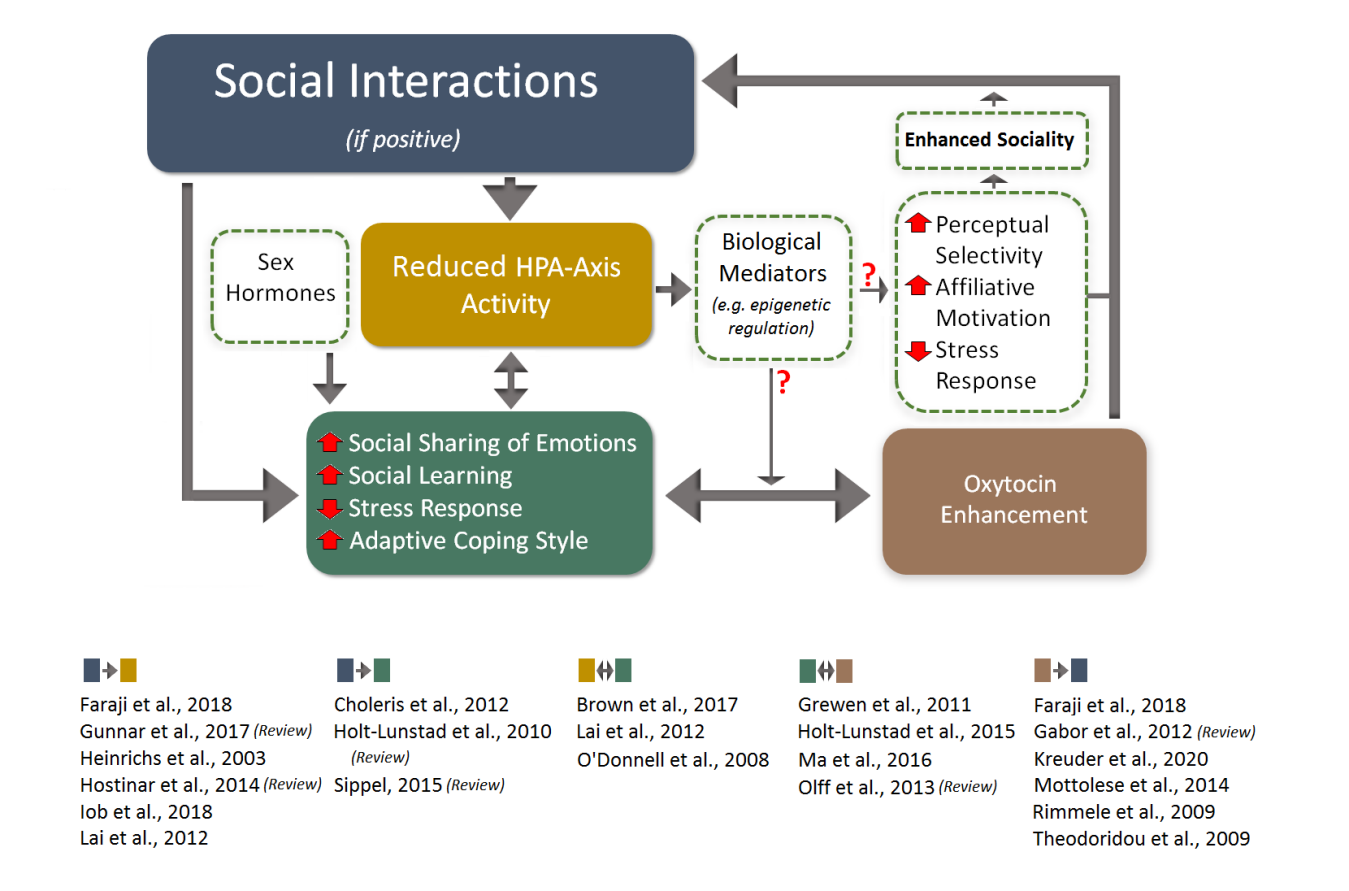
Representation of hypothetical mechanisms that potentially affect the impact of social relationships in humans. Positive social relationships are characterized by reduced HPA axis activity, which translate into positive emotions in a sex-dependent manner. Via biological mediators, such as epigenetic regulation, and via expression of the peptide hormone oxytocin, both the HPA axis and the positive emotional state will affect the resilience, coping and mental wellbeing, and ultimately promote healthy aging and longevity.

Interestingly, it appears that males and females, both human and animal, respond differently to social interactions [91]. These differences in response to social cues may range from changes in neurobiological measures and processes [42, 92-94] to displaying distinctive sex- and gender-specific behaviours [91, 95-97] across the life course. Sharply different levels and lifetime profiles of sex hormones (e.g. androgens and estrogens) lead to striking differences between male and female behaviours [98, 99]. Of all sex-related behavioural disparities, sex differences in social behaviour is one of the most pronounced. Major aspects of sex differences in social interaction may be explained by the response to oxytocin (OT), an evolutionarily conserved neuropeptide which is critically involved in social bonding, cognition and improved coping with stress [100, 101]. For instance, females appear more vulnerable than males to genetic and behavioural changes when the secretion of endogenous oxytocin in response to social life is interrupted [102]. Also, oxytocin may facilitate various forms of prosocial behaviours, building trust and enhancing sociality in humans and non-human animals [103-109], an effect that would presumably enhance social engagement (Fig. 2). The interaction between oxytocin and sex hormones, especially estrogens, also appear to impact social recognition and regulate social learning [110, 111], the main foundations of a social interactions. While the first enables an individual to distinguish between conspecifics and to establish close social relationships with a familiar being, the latter provides a process through which the individual learns to observe a conspecific and/or decide how to interact with others.

In line with what has previously been established in female rodents [112], estrogenic receptor (*ESR1* and *ESR2*) signals may regulate social recognition in women [113]. Androgens, on the other hand, may play a role in the male-biased function of neural circuits that develop specialized roles in social relationships [114]. For example, testosterone, a primary male sex hormone that works directly via the androgen receptor (AR) and is known as an inhibitor of sociality, significantly decreases interpersonal trust when administered to females [115]. In addition to the endocrine pathways, social interactions affect human development and aging also via the major biological domains of the hypothalamic-pituitary-adrenal (HPA) axis and the immune system.

### Social Interaction and HPA Axis Activity

A general assumption is that there are functional perturbations with advancing age that result in dysfunctional activity of various endocrine glands and their target organs [116, 117]. These hormonal challenges in the elderly also may interfere with hormonal cross-communication between various endocrine axes. Accordingly, the neuroendocrine theory of aging [118, 119] refers to the consequences of age-related changes in the HPA axis, the key adaptive neuroendocrine system that responds to stress. Cumulative evidence from human and non-human studies suggest that the HPA axis is altered by aging [116, 120-124]. The HPA axis is also subject to diurnal variation and represents a neurohormonal hallmark of emotionality in humans and animals [91, 125]. HPA-axis activity is characterized by prominent sex differences as females initiate it more rapidly in response to stress and produce a greater output of stress hormones [126-128]. In general, sex differences in HPA-axis activity are evident already early in life [129, 130] and reveal higher baseline glucocorticoid (GC) levels and higher absolute GC concentrations during stress in females than males. GC levels also remain elevated for longer in females, suggesting an increased stress reactivity in females along with reduced negative feedback. Importantly, when long-term stress exposure (e.g. disease, trauma, isolation and loneliness) is inevitable, it appears that females display more flexible and adaptive behavioural responses than males [131, 132]. Therefore, it seems justified to conclude that, influenced by the hormonal changes of menopause in women, the aging brain also shows both sex-specific responses to stress and sex-specific capacity of building resilience.

The activation of the HPA axis and elevated circulating GCs are essential for adaptive responses to acute and chronic stress and thus elemental for the individual’s survival, even though its dysregulation is apparent during aging. Because complications in HPA axis activity in the elderly begin with high GC levels, experimental challenges have highlighted the impaired GC-mediated inhibitory (negative) feedback loop and reduced GC receptor sensitivity in the brain [133]. Both conditions lead to prolonged HPA axis activation and a long-term elevation in GC in the aging brain [120, 133, 134]. Another key aspect of functional alterations in the aging brain is the loss of GC receptors in the hippocampus (HPC), a sexually dimorphic structure intimately involved in learning, memory and spatial navigation. Interestingly, the hippocampus shows the highest density of corticosteroid receptors in the brain [135] and critically contributes to the negative feedback to the HPA axis, making sure the stress response shuts down after cessation of stress [136, 137]. Hence, because the hippocampal volume decreases with aging, the consequent weakening of negative feedback may be responsible for HPA axis dysregulation and over-activity.

Although this topic falls beyond the scope of the present review, it is necessary to state that prolonged disturbances in HPA axis function are closely tied to psychophysiological pathologies in elderly, such as higher risk of cognitive decline [138-140], depression [141, 142], and anxiety and stress-related disorders [143, 144]. Moreover, aging-associated HPA axis dysregulation raises the risk of cardiovascular diseases and hypertension [145], stroke [146] and diminished immunity [147]. Altered HPA axis functioning in old age may also critically contribute to degenerative pathologies, such as Alzheimer’s disease (AD) [138, 148, 149] and Parkinson’s disease [150]. Using a mathematical model in humans, McAuley and others showed that both acute (transient) and chronic (repeated) elevations in cortisol secretion increase aging-associated HPC atrophy and loss of hippocampal activity [151]. Even though transient activation of the HPA axis is essential for stress adaptation, prompt termination of the stress response is necessary to prevent deleterious consequences of excessive GC levels. On the other hand, the normal stress response can be profoundly impacted by chronically elevated GC levels in the elderly, leading to withdrawal and avoidance [152], impaired ability to recover from stressful stimuli [117], and consequently accelerated aging [153].

Health-related concerns linked to an overactive HPA axis also emerge from the sexual dimorphism observed in humans [154] and non-human animals [91, 155].Females typically show more variability in HPA axis activity than males [155-158]. Due to prolonged GC elevation in the aging brain, chronic stressful experiences increases the risk for stress-related pathology mostly in women [159]. Clinical efforts therefore focus on the patterns of sex- and age-related changes in HPA axis activation as well as how to reduce vulnerability to stress-related disorders during aging [119].

It has been widely recognized that HPA axis activity can be regulated by a socially stimulating environment [16, 59, 160-162]. For instance, social relationships, if positive, can dampen HPA axis stress responses across the lifespan, from early development [163], throughout adulthood [164] and into old age [165, 166]. Importantly, elderly individuals who are more involved in developing and strengthening their social relationships show more effective activation and deactivation of the HPA axis [166]. The stress-buffering effect of social relationships was also explored in rodents with results that have been reliably consistent with clinical findings [67]. Prolonged social experiences in rats may form new biobehavioural phenotypes such as reduced HPA axis activity, not only in directly exposed individuals, but also in their unexposed (non-social) descendants in a sexually dimorphic manner [91]. Social enrichment in animals promotes expression of glucocorticoid receptors [167] and mineralocorticoid receptors [168] in the brain, thus enhancing effective HPA axis regulation and negative feedback function.

Socially engaging experiences also appear to promote healthy aging via OT and brain-derived neurotrophic factor (BDNF) regulation. In fact, the benefit of social support was causally linked to elevated OT levels, but TL became elongated only in females. Interestingly, the OT antagonist L-366,509 blocked all benefits of social housing [91, 102]. It was also recently shown [169] that chronic social isolation leads to increased plasma CORT and cellular aging in aged prairie voles. However, daily OT injection can prevent these adverse consequences of social isolation. Along with improved HPA axis regulation by social support, BDNF also seems to be an alternative key pathway that mediates positive consequences of social context. Upon activation by OT, BDNF may engage neuronal survival mechanisms and promote resilience at the neuronal level [91, 102]. Thus, prolonged social interaction promotes neuroplasticity and stress resilience particularly in females, via OT and BDNF regulation, while OT deficiency exacerbates stress vulnerability. In fact, OT was proposed as an ideal anti-aging agent because it promotes stem cell proliferation and regeneration [170], but these effects were not yet tested in the brain, and the link between OT and BDNF is still unclear.

### Social Interaction and Immune System Function

Older adults are at higher risk for COVID-19 complications than the younger population, partially due to a weaker immune system and potentially other pre-existing health conditions [171]. Aging is associated with profound physiological changes that also affect the immune system, a complex host defense system that protects the organism from pathogens, either internal or external. Immune changes with aging, commonly known as immunosenescence, mainly involve a decline in many immune parameters when compared to young healthy individuals [172]. The immunosenescence and a chronic low-grade inflammation called inflamm-aging [173, 174] stand together at the origin of most diseases of the elderly. The age-related immune decline typically refers to the gradual deterioration, profound remodeling and alterations of immune function with major impact on health and survival during senescence [172, 173, 175].

The immune system, which acts in close dialogue with the neuroendocrine system including the HPA axis [176], also exhibits significant sex-specific differences [177, 178]. Aside from age-specific changes in immune function, men and women also respond differently to invading pathogens. This sexual dimorphism in immune response may have a serious impact on, for instance, the magnitude of inflammatory responses and pathogenesis of many infectious diseases that are a major cause of mortality among older adults. An increase in inflammatory pathways and immune-mediating genes and a decline in B-cell specific loci has recently been reported in older males [178] which suggests an accelerated inflamm-aging trajectory in aging men. At comparable exposure levels, men also seem to be more susceptible to infections caused by viruses, bacteria, parasites, and fungi [179]. Hence, sexually dimorphic susceptibility to inflamm-aging and infectious diseases, along with dysregulated adaptive immunity and increased systemic inflammation in the elderly, require careful consideration in age-specific interventions and therapies.

The molecular and cellular foundations of immunosenescence are still unknown. A growing body of evidence, however, has attributed the age-related immune decline to overproduction of pro-inflammatory cytokines such as interleukin 1β (IL-1β), interleukin 6 (IL-6), interleukin 18 (IL-18), C-reactive protein (CRP), and tumour necrosis factor alpha (TNF*a*) in the innate immune system [180-186]. Specifically, increased production of IL-6, a key pro-inflammatory cytokine, appears to be associated with a range of immune-mediated conditions in the elderly including rheumatoid arthritis, osteoporosis, atherosclerosis, type 2 diabetes, cardiovascular disease, AD, some forms of cancers, and frailty [182, 187-192]. Recently, it was shown that elevated cortisol and plasma CRP levels in a large sample of older adults were associated with more persistent depressive symptoms over a 14-year period [193]. These aging-related diseases occur in an inextricable relation to inflammation and HPA axis dysregulation, and may enhance general disability [194] and mortality among older adults [195, 196]. In turn, good overall health in old age may be the direct consequence of a low pro-inflammatory state [188]. However, the aging phenotype, including immunosenescence and its immunological correlates, although unavoidable, may be mitigated through psychological interventions that support immune function and healthy aging.

Immune changes that occur during the aging trajectory are not uniform, however, involving down-regulation of some functions and up-regulation of others. Whether immune functions increase or decrease during aging depends also on psychological influences [147, 197-201], such as positive social interactions that may promote healthy aging. A growing body of literature has linked alterations of inflammatory measures in late life to the quality of social connectedness [37, 202-204]. It was suggested that perceived positive relationships and social integration may be associated with lower IL-6 and higher CRP in older adults, particularly in women [205]. It should be pointed out that the association between CRP and social relationships is still lacking consistency [203], arguably due to differences in sociocultural structures [206]. Although inflammatory markers in both sexes appear susceptible to the quality of social relationships [37], social support in particular can reduce IL-6, especially in older women [205]. In general, it seems that structural dimensions of social connectedness are more related to inflammation (particularly CRP) in older men, whereas functional dimensions of social relationships have an impact on inflammation (particularly IL-6) in older women [205, 207].

In an alternative approach to the psychological determinants of immunological responses and disease susceptibility [208], it was recently suggested that chronic psychological stress is among factors that can be associated with greater risk of respiratory illnesses after a bacterial or viral exposure (e.g., cold or influenza), and potentially after COVID-19 infection. Less association, however, was observed among participants who reported optimal social integration and support. It was, therefore, proposed that the pathways linking psychosocial factors (e.g., psychological states and social interactions) to colds and influenza may play similar roles in COVID-19-related complications. The general similarities seen among the symptoms of cold, influenza and COVID-19, such as pro-inflammatory cytokine up-regulation in disease pathogenesis along with psychosocial mediators that may predict disease susceptibility and severity support the idea that these immune challenges may impact overall health and resilience through common pathways [208].

### Social-physical distancing and isolation: solution or problem?

In spring 2020, health agencies and governments around the globe have recommended lockdown and physical distancing in order to contain the spread of COVID-19. These measures have been widely successful in epidemic control by limiting the exposure to the general population and reduced (by 45%) COVID-19 transmission and associated long-term health risks (for instance, see [45]) and significantly reduced fatality [4]. At the same time, others have cautioned that physical distancing and confinement may also exacerbate existing social issues. For example, Michael Levitt, a Nobel laureate biophysicist at Stanford University, has challenged that “*lockdown [for COVID-19] caused more deaths than it saved*” through “*social damage - domestic abuse, divorces, alcoholism*” in an interview with the *Telegraph* on May 23^rd^, 2020 (https://www.telegraph.co.uk/news/2020/05/23/lockdown-saved-no-lives-may-have-cost-nobel-prize-winner-believes/). The period of physical distancing during COVID-19 may have therefore exposed the most vulnerable individuals and populations and revealed where social support and interventions are most urgently required.

### Aging and Social Disengagement: Challenges and Complexities

Elderly people are among the most vulnerable individuals of our society. As people age, they become increasingly susceptible to adverse consequences of social-physical distancing and withdrawal. This may lead to gradual reduction in elderly individuals interacting with their social environment, thus shrinking their social network. Also, a growing global gender gap in the proportion of elders (over 60) who reside alone (17% of women vs. 9% of men) has been reported [209]. In the USA, nearly every second woman (45%) aged 75 and over lived alone in 2017, and approximately 28% (9.3 million women, 4.5 million men) of non-institutionalized older individuals lived alone, unveiling a hidden sex-specific trend of social disengagement and loneliness in aging (https://acl.gov) [210]. Yet, by implementing social contact restrictions, the rapidly evolved COVID-19 outbreak has further changed the conventional faces of social communication for all ages throughout the world [1]. Older adults have been especially affected by this double-edged sword; on one hand, this vulnerable group was better protected against COVID-19 risks, on the other hand, they lost important face-to-face interaction and personal attention at a time when support was critically needed. Thus, the COVID-19 pandemic may particularly affect older adults who are dependent on care. Contact restrictions during a pandemic therefore have the potential to generate the perception of social isolation and loneliness, which may raise the susceptibility to further long-term mental and physical health challenges. Moreover, events such as shrinking economic resources, mobility impairment, and the death of relatives or friends may reduce the size of a social network during aging, and consequently accelerate age-related health problems [21, 51, 204, 211-213]. As discussed earlier, individual differences in stress response and resilience are determined not only by sex, but also by age [214]. In experimental studies, social isolation affects young and aged rats differently, depending on the age at isolation and its duration [215]. In humans, social isolation may exacerbate stress response amongst elderly and contribute to anxiety, depression and post-traumatic stress disorder [46]. These findings highlight the vital role of social support in healthy brain aging and the timely need for social neuroscience approaches.

### Social Isolation and Loneliness in Aging

Social isolation is typically referred to an objective state of reduced social contact and networking [86] or disengagement from social ties and participation [216], mostly due to environmental restrictions or physical demands [217]. In fact, when experiencing social isolation, an individual lacks a sense of belonging or engagement with others [218]. Social isolation represents the quantitative absence of social connectedness, whereas loneliness is the extent to which an individual subjectively (or emotionally) feels himself or herself socially isolated even when among other people [20, 36, 219]. Loneliness, although conceptually distinct from social isolation, represents the psychological embodiment of social isolation [86]. It is noteworthy that “being alone” and “feeling alone” should not be confused [220], even though both appear to be common problems of old age. Biological responses to stress may increase physical susceptibility (e.g., via dysregulation of the HPA axis and/or immunity) to psychological difficulties and ultimately disease, thus targeted interventions to mitigate social isolation and loneliness can significantly improve quality of life and long-term health outcomes [221].

The concept of social isolation and loneliness portrays a profile of profound impact on mortality in older adults, either synergistically [222, 223] or independently [224, 225]. Also, it seems that each involves a distinct pathway through which they influence health risk factors [86, 219, 226]. While social isolation is linked to physical or general health, the loneliness has an impact on mental health [225, 227, 228]. Thus, health practitioners need to consider and assess social isolation and loneliness simultaneously, even though interventions for social isolation appear to be very different from interventions against the feeling of loneliness.

Aside from correlational investigations linking social exposure to psychophysiological health in later life [37, 60, 66, 165, 229, 230], a wide range of socially supportive interventions have also been utilized to effectively tackle social isolation and loneliness, thus reducing their deleterious impact on health outcomes [231-236]. Although these efforts have been shown to promote social engagement, interventions that focus merely on building social networks often fail to mitigate perceived social isolation [220]. Healthcare providers should be aware that there is no one-size-fits-all approach for addressing social isolation and loneliness. Instead, any intervention to mitigate their harmful consequences necessitates a precise assessment of personal needs, taking into account their gender, ethnicity and culture and the intensity of isolation or loneliness experienced [237].

Even if health professionals and policy makers consistently follow up with the above concerns, there are also further complications that need to be seriously addressed. For one, social isolation and loneliness in later life represent a geo-specific dynamic impact on health and regional interplay, most likely due to the influence of sociocultural determinants. While these two are not highly correlated in the USA and UK [86, 225], and with no synergistic effect in Finland [224], both have been closely interacting in relation to mortality in Germany [222]. In addition, compared with loneliness, efforts to reduce social isolation are likely to be more relevant and to have greater benefits in terms of mortality in UK [86, 238], but having fairly opposite effects in the USA [36]. In the Netherlands, conversely, only loneliness was found to be a major risk factor for increased mortality in older individuals, and more men than women who felt lonely died during a 10-year follow-up [239]. However, this disparity may be generated by differences in assessment policy and monitoring, along with variations in conceptualization of social isolation and loneliness across cultures and societies. Interventions tailored to identifying and ameliorating the harmful consequences of social isolation and loneliness must contemplate not only how to conceptualize and measure them, but also the normative values of the respective community and cultural framework that typically determine the purpose and meaning of life in older people [240]. More importantly, the most effective interventions imply adaptability of the intervention to a local context within a community development approach [241]. Practitioners and policy makers, therefore, need to ensure that they understand respective community and culture, and form a context-sensitive intervention in tackling social isolation and loneliness in later life.

### Aging, social distancing, and covid-19: the two-hit model of stress

In addition to cultural measures, the individual’s past experiences and even that from previous generations may generate complex interaction and synergies with the biological aging process and social life [10, 25, 242]. Also, this relationship is strongly influenced by pre-existing health conditions, gender, personality and culture [243-245].

Stress, in this review, refers to endocrine and behavioural responses to stressors that perturb homeostasis in a number of physiological systems (e.g., anxiety, body temperature, osmolarity, oxygen tension) [246, 247]. The suite of these responses is essential to survival, but may not necessarily adapt the organism to its environment. Responses to repeated stress, however, are intended to maintain homeostasis by maximising adaptability, resilience, or allostasis. Allostasis mediated by the immune system, autonomic nervous system (ANS) and the HPA axis refers to a process through which the body adaptively changes to maintain stability in the face of stressors (e.g. diseases, insults, hazards, injuries *etc*.) [26, 247]. Allostatic responses to repeated stressors, although advantageous in the short run, impose a cost for adaptation: chronic allostatic activation results in a permanent physiological shift with abnormal vital values when stress hormones and inflammatory mediators are frequently mobilised in the attempt to maintain homeostasis and maximise resilience.

### Allostatic Load in Elderly: Concealed Aspects of the Chronic Stress Impact

Allostatic load (AL), in contrast, reflects the body’s “wear and tear” when the mediators of allostasis fail to promote resilience but rather accumulate the biological burden associated with repeated stressful hits [248]. Notably, the aging trajectory reflects the cumulative impact of wear and tear previous ancestral, prenatal and lifetime psychological, physical and inflammatory stressors, with each stress representing a “hit” [23, 249, 250]. High AL or unproductive resilience to stressors with cumulative wear and tear induced by multiple stress hits seems a hallmark of aging in a gender-based trajectory [251, 252], reduces longevity [253], and ultimately predicts frailty, morbidity and mortality [254]. Moreover, stress-associated immune modulation may also increase the risk of infectious diseases in elderly [255], and these health outcomes may be exacerbated by stress induced through social isolation or loneliness.

**Figure 3.**
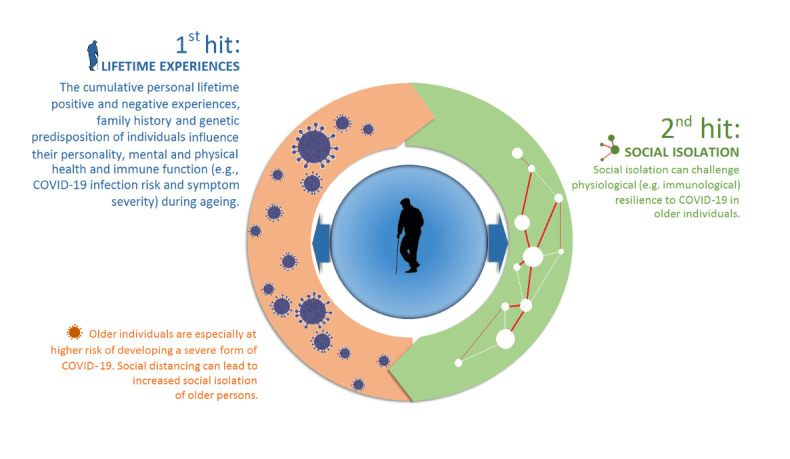
The two-hit model of vulnerability to COVID-19 infection in older individuals. According to the two-hit model of stress, developmental or lifetime stress exposure represent the first hit that makes an older individual more vulnerable to experiencing a second hit, such as social isolation, as being stressful. The cumulative impact of perceived stress may potentially reduce the resilience when faced by a COVID-19 immune challenge.

Cumulative wear and tear by recurrent stresses may raise the risk of age-related physiological complications and the susceptibility to adverse health outcomes [256]. Loss of social relationships and confinement during a pandemic may further challenge the psychophysiological resilience in elderly [37] and reduce the protective benefit that comes with social enrichment [66, 257]. Each of these stressors may represent a hit that challenges the delicate balance between allostatic mediators of the stress response such as adrenal hormones, inflammatory cytokines and neurotransmitters [24]. In this context, the two-hit model of stress would suggest that developmental or lifetime stress exposure (first hit) makes an older person more vulnerable to a subsequent perceived stressor (second hit), such as social isolation, and this may reduce the resilience when faced by a COVID-19 immune challenge (Fig. 3). Thus, the cumulative impact of recurrent stresses throughout a lifetime might challenge the immunological tolerance or resilience to the COVID-19 virus and increase the risk of morbidity and mortality due to the infection. While the first hit may induce a permanent physiological shift away from the normal homeostatic range in the attempt to adapt, the second hit may disrupt this fragile allostatic equilibrium and diminish resilience to disease [253, 258]. Hence, the combined impact of the two stressors is more influential than either hit alone [23, 25]. This concept is ultimately dependent upon the individual’s perception and cognitive appraisal of a challenging situation, however, and social isolation may not necessarily be perceived as a stressor.

### Mitigation strategies and interventions

The COVID-19 pandemic and the required social contact restriction for epidemic control have challenged daily routines of social life in the elderly population, such as restricted contact with family members, friends and even care providers for an extended period of time. Future research will provide evidence if the design and development of targeted social interventions may provide effective relief from the impact of COVID-19-related social isolation and loneliness. Previously designed social interventions for older adults showed that they can be effective when provided as one-on-one assistance [241]. Such interventions may be implemented through information technology (IT) such as video conferencing and social media [232, 259], or solitary pet ownership and animal-assisted intervention [260-262]. Also, a short term (2-week) smartphone-based mindfulness training for reducing emotional social isolation improved social engagement in older adults [263]. Hence, although contradictory to the current trend that emphasizes building social networks through group settings, solitary interventions focusing on individual activities and engagements, may successfully assist individuals to combat objective and subjective isolation. Indeed, when a growing number of individuals are unable to easily participate in group-social activities, solitary context-sensitive interventions may make a meaningful, practical and cost-effective difference. Notably, the considerable diversity of the aging population, in particular the sex-based variations in response to social isolation stress and lifestyle, require caregivers, social service professionals, and community authorities to consider the salience of individual differences when designing and implementing supportive interventions.

Experimental studies have also been able to specifically study the impact of social interactions on long-term health and aging. For example, environmental enrichment (EE) by housing laboratory rodents in social groups has become a widely used paradigm that effectively reduces the chronic burden of stress and promotes long-term health and aging [264]. This research has also shown that females are generally more responsive to social aspects of EE than males [16, 91, 102]. These benefits are associated with oxytocin-mediated telomere elongation, a biological marker of longevity, indicating improved aging trajectories [102]. This work has helped elucidate the biological underpinnings of social isolation and support the critical role of social enrichment as a treatment option to reverse adverse consequences of stress and enhance stress resiliency.

### Conclusion and synthesis

The global COVID-19 outbreak in 2020 has fundamentally changed ways of social interaction and communication at all levels and at all ages. The impact is being particularly felt in the aging population and has called for intergenerational solidarity. Staying socially engaged is a critical part of successful aging, and staying connected is a vital part in combating loneliness, anxiety and depression [51, 230]. Maintaining meaningful social interactions offers many benefits to elderly, including reduced risk of Alzheimer’s and Parkinson’s diseases [265, 266], improved cognitive skills [266, 267], lower blood pressure and cardiac health [37, 268], lower incidence of infectious diseases [269], and greater longevity [43]. Insights into how social support enhances allostasis and resilience to stress offers insights into the underlying links between psychosocial and physical health, with important clinical relevance to predicting and preventing adverse health outcomes within a personalized medicine framework. The adverse consequences of psychosocial stress and anxiety around social isolation on physical health in the elderly should not be underestimated. The COVID-19 pandemic has markedly exposed the vulnerable situation of our society’s aging population. Here, social-physical distancing, if not implemented correctly and reasonably, may act as a double-edged sword, and impose more costs to an aging society through increasing mortality and morbidity. This situation highlights the urgent need for further research into experience-dependent determinants of aging trajectories and the need for development and implementation of practical and accessible interventions. At the same time, the COVID-19 pandemic also presents an unprecedented opportunity to raise public awareness of vulnerable populations and create resources for building resilience for our future and the next generations.
